# Comparison between flutter valve drainage bag and underwater seal device for the management of non-massive malignant and paramalignant pleural effusions

**DOI:** 10.11604/pamj.2020.35.3.19197

**Published:** 2020-01-07

**Authors:** Olugbenga Olalekan Ojo, Martins Oluwafemi Thomas, Ezekiel Ogunleye, Olugbenga Olusoji, Uvie Ufuoma Onakpoya

**Affiliations:** 1 Department of Surgery, College of Health Sciences, Obafemi Awolowo University, Ile-ife, Nigeria; 2 Department of Surgery, Lagos University Teaching Hospital, Lagos, Nigeria; 3 Department of surgery, College of Medicine, University of Lagos, Idi-araba, Lagos, Nigeria

**Keywords:** Malignant, paramalignant, pleural effusion, flutter valve, underwater seal

## Abstract

**Introduction:**

The aim of this study is to compare the use of flutter valve drainage bag system as an alternative to conventional underwater seal drainage bottle in the management of non-massive malignant/paramalignant pleural effusion.

**Methods:**

Forty-one patients with non-massive malignant and paramalignant pleural effusions were randomized into two groups. Group A (21patients) had their chest tubes connected to an underwater seal drainage bottle, while group B (20 patients) had their chest tubes connected to a flutter bag drainage device. Data obtained was analyzed with SPSS statistical package (version 16.0).

**Results:**

Breast cancer was the malignancy present at diagnosis in 24(58%) patients. Complication rates were similar, 9.5% in the underwater seal group and 10 % in the flutter bag drainage group. The mean duration to full mobilization was 35.0±20.0 hours in the flutter bag group and 52.7±18.5 hours in the underwater seal group, p-value 0.007. The mean length of hospital was 7.9±2.2 days in the flutter bag group and 9.8±2.7 days in the underwater seal group. This was statistically significant, p-value of 0.019. There was no difference in the effectiveness of drainage between both groups, complete lung re-expansion was observed in 16(80%) of the flutter bag group and 18(85.7%) of the underwater seal drainage group, p-value 0.70.

**Conclusion:**

The flutter valve drainage bag is an effective and safe alternative to the standard underwater seal drainage bottle in the management of non-massive malignant and paramalignant pleural effusion.

## Introduction

Thoracic drainage systems are designed to remove air and fluid from the pleural space [1] and the underwater seal drainage system in its present form was first described by Kenyon in 1916 and since then has been the standard form of chest drainage [2]. Placement of underwater seal below the chest wall often causes disconnection of the connectors [3] and it also puts tension on the anchoring stitch, resulting in increased risk of chest tube mishaps. The frequent clamping during transport can cause pulmonary collapse, formation of clots and can result in worsening of an existing pneumothorax [3]. In 1968, Henry Heimlich idealized a device (The Heimlich valve) to replace under water-seal drainage systems [3] and this is the concept of the flutter valve drainage bag as popularized by Portex USA. Thompson *et al.* [2, 4], showed that bags with integral non-return valves could be used for chest drainage. The flutter valve drainage bag is a ‘no water’ ambulatory system which incorporates a one-way valve and can be used both in hospital and outpatient drainage of pleural collections [5]. The one-way valve system provides better mobility of patients because clamping is unnecessary during transportation and the valve keeps working regardless of its position/level [3]. Vuorisalo *et al.* [6] showed that the flutter drainage system is a safe and feasible system when pleural drainage is needed in the treatment of pneumothoraces and pleural effusions.Pleural effusion is an abnormal accumulation of fluid in the pleural space and neoplastic diseases accounts for 13% to 40% of all the pleural effusions worldwide and account for 70% of all massive effusions [7]. Ogunleye *et al.* [8], also found that neoplastic diseases were the commonest cause of pleural effusions. This was similar to what was found earlier by Thomas *et al.* [9] in Lagos, Nigeria.

Malignant pleural effusion is confirmed by the presence of malignant cells in pleural fluid or tissue [10]. In patients with established malignancy and pleural effusion, when malignant cells have not or cannot be detected in the pleural fluid or tissue, Sahn *et al.* [10] labels this group as paramalignant effusions. Malignant Pleural Effusion (MPE) complicates the course of various malignancies, with most cases occurring secondary to pleural metastasis of lung and breast adenocarcinoma [11, 12]. Malignant pleural effusion is the commonest indication for insertion of chest tube and drain in Lagos Nigeria and a great number of such individuals are females with breast cancer [8, 9]. Massive pleural effusion can be defined on plain radiograph as, complete opacification of an entire hemithorax with or without mediastinal shift [13]. Thus pleural effusions that do not fulfill this definition can be regarded as non-massive pleural effusion. Patients with non-massive malignant or paramalignant effusions end up with underwater seal drainage devices after chest tube insertion. These patients often compete for limited bed space and they are hospitalized for at least a week. They are rendered immobile and their tubes get blocked from repeated clamping during transport with increased risk of iatrogenic pneumothorax and hospital-acquired infections. This study intends to compare the use of flutter valve drainage system as a safe and useful alternative to conventional underwater seal drainage in management of non-massive malignant and paramalignant pleural effusions.

## Methods

Patients with non-massive malignant or paramalignant pleural effusions admitted into the Lagos University Teaching Hospital (LUTH) from January 2014 to December 2014 were prospectively enrolled in the study. Approval was obtained from the Hospital's Health Research and Ethics Committee. Forty-one patients presenting with non-massive malignant and paramalignant pleural effusions were consecutively divided into two groups. Group A (21 patients) had their chest tubes connected to an underwater seal drainage bottle, while group B (20 patients) had their chest tubes connected to a flutter bag drainage device. All consenting adult patients (≥ 18years) with non-massive malignant and paramalignant pleural effusions were considered eligible for inclusion in the study. Individuals with effusions not due to or related to a malignancy, massive pleural effusion and complicated effusions (recurrent effusion, trapped lung/empyema thoracis) were excluded from the study. After obtaining informed consent, patients in both groups had a size 28F (French size) chest tube inserted aseptically under local anesthesia, via the 6^th^ intercostal space mid axillary line and anchored to the skin with appropriate size non-absorbable suture.

Patients in Group A had their chest tubes connected to the standard underwater seal drainage system while Group B was connected to the flutter bag drainage system (Portex). All patients had administration of prophylactic antibiotics and adequate analgesia. A post insertion chest radiograph was obtained in all patients to confirm proper placement of chest tubes and daily drainage via chest tube was also noted. Drainage was discontinued and chest tubes were removed once daily output became ≤ 100ml/24hours for 2 consecutive days (in the presence of a patent tube) and chest radiograph showed re-expansion of the underlying lungs. Post chest tube removal radiographs were obtained and the patients were discharged home for follow up in clinic. Comparison was done between the two methods of chest drainage based on the selected parameters. Statistical analysis was conducted by using the SPSS 16.0 for Windows program (Chicago,SPPS Inc.). A P-value of < 0.05 using the Fishers exact test was considered significant.

## Results

Of the 41 patients recruited for the study, there were 37 females (90.2%) and 4 males (9.8%) in the study population with a mean age of 50.3±14.3 years (Table 1). Breast cancer was the commonest malignancy present at diagnosis of pleural effusion in 24(64%) of the 37 female patients, which translates to 58% of the entire study population. Endometrial cancer was present in 8(21%) of the 37 female participants and this corresponds to 19.5% of the total population (Table 2). Out of the 4 male participants, lung cancer was present in 2 (4.9% of the total population), the third male patient had osteosarcoma of the lower limb and the fourth was a case of mediastinal non-seminomatous germ cell tumor. The mean volume of pleural fluid drained was, 1800±633mls in the flutter bag group and 1,740±664mls in the underwater seal group. There was a statistical significance in the duration to full mobilization of patients between both groups, 35.0±20.0 hours in the flutter bag group and 52.7±18.5 hours in the underwater seal group, P-value 0.007 (Table 3).

**Table 1:** Distribution of patients with age ranges in decades

Age ranges in decades	Number of patients	Percentage (%)
<20	1	2.4
21-30	1	2.4
31-40	10	24.4
41-50	10	24.4
51-60	8	19.5
61-70	8	19.5
71-80	3	7.7
**Total**	41	100

**Table 2:** Type of malignancy at diagnosis of pleural effusion

Malignancy at Diagnosis	Frequency	Percentage (%)
Breast	24	58.5
Endometrial	8	19.5
Hepatic	2	4.9
Lung	3	7.3
Mediastinal germ cell tumor	1	2.4
Osteosarcoma	1	2.4
Ovarian	2	4.9
**Total**	41	100

**Table 3:** Comparison of duration to full mobilization

Group	Number	Mean duration to full mobilization (hours)	Standard deviation	Standard error mean	p-value for significance
Flutter bag	20	35.0	20.9	4.7	
Underwater seal	21	52.7	18.5	4.1	P-value 0.007

*P-value* = 0.007(using Fishers exact test)

There was no significant difference in the mean duration of drainage between both groups, 6.5±2.6 days in the flutter bag group and 7.9±2.0 days in the underwater seal group, p-value 0.059 (Table 4). However, there was a significant difference in the length of hospital stay, with the patients in the underwater seal group having a longer hospital stay (P = 0.019) (Table 4). There was no difference in the effectiveness/completeness of drainage between both groups as seen in the comparison of pre and post extubation chest radiographs. There was also no difference in complication rates between the two groups studied. Overall there were 4 (9.8%) complications in the entire study group. 2 in each group, which translates 10% in flutter valve bag vs 9.5% in the underwater seal group (Table 5).

**Table 4:** comparison, showing duration of drainage and length of hospital stay in the study groups

Variables of patients	Patient groups	Mean	Standard deviation	Standard error mean	p-value for significance
Duration of drainage(days)	Flutter bag	6.5	2.6	0.6	*0.059
	Underwater seal	7.9	2.0	0.4	
Length of hospital stay	Flutter bag	7.9	2.2	0.5	**0.019
	Underwater seal	9.8	2.7	0.6	

* *P* value >0.05.

** *p* value <0.05

(Fishers exact test)

**Table 5:** Comparison of complication rates between groups

Complications	Group			
	Flutter valve bag	Underwater seal	Total	p-value
Tube blockage	1	1	2	
Wound infection	1	1	2	*1.00
None	18	19	37	
**Total**	20	21	41	

* *P*-value 1.00(Fischer exact test)

## Discussion

The underwater seal drainage bottle has been the conventional reservoir for drainage of pleural effusion in most patients, however various studies including that of Graham *et al.* [2] and Vourisalo *et al.* [6] have demonstrated that, the flutter valve drainage bag may be a useful alternative to the conventional under water seal bottle. Malignant and paramalignant pleural effusions pose a lot of challenge and constitute a huge burden to patient’s physical, social, mental and financial wellbeing. Malignant diseases cause 13% to 40% of all the pleural effusions worldwide and account for 70% of all massive effusions [7]. In this study, the commonest malignancy present at diagnosis of pleural effusion was breast cancer (24 patients (58%)), which was similar to the findings of other authors [7-9]. There was a significant decrease in the number of hours from sitting to full mobilization in the flutter valve drainage bag group, when compared to the underwater seal group (P-value 0.007). This supports the findings of Graham *et al.* [2] and further lends credence to the likelihood of reduction in the incidence of deep vein thrombosis in the flutter bag group, due to early mobilization. There was no difference in the duration of drainage when employing the flutter bag drainage system or the underwater seal drainage device in this study which is confirmed by a previous work [2]. The lack of significance in duration of drainage when using an underwater seal device or a flutter valve drainage bag was also demonstrated in studies by Vourisalo *et al.* [6] Although one can argue that the underwater seal group in their study, had their chest tubes connected to a suction device, this did not seem to affect the overall significance.

The patients in the flutter valve drainage bag group had a shorter period of hospital stay when compared to the underwater seal group and this was statistically significant with a P value of 0.019. This contrasts with the works of Graham and Vourisalo, but it is in keeping with the findings of Kadkhodaei *et al.* [14]. The difference in significance might be because the individuals in the earlier studies were post-operative patients and may have other confounding factors that resulted in their extended hospital stay. In this study, the completeness of drainage based on plain chest radiographs obtained after chest tube removal, showed complete lung expansion in 16(80%) of the flutter bag group vs 18(85.7%) in the underwater seal group. There was incomplete lung expansion in 4(20%) of the flutter bag group vs 3(14.3%). However, further drainage was required in 2 patients in the flutter bag group as opposed to 1 patient in the underwater seal group. This was similar to the findings of Vega *et al.* [3], in their evaluation of 36 patients who had chest drainage with flutter valve bags post lung resection.

Vega *et al.* [3] in their study showed that, the chest radiographs obtained after removal of flutter bag drainage system were considered normal in 26 patients (72.8%), whereas incomplete lung expansion was seen in 8 patients (22.4%) and small pleural effusion was seen in 1 (5.6%). The main difference between this study and that of Vega *et al.* [3] is that theirs was not a comparative study. The occurrence of complications was similar in groups, 10% in the flutter bag group and 9.5% in the underwater seal group. This was similarly demonstrated in the Graham *et al.* study, where the incidence of complication was 11(17%) in the flutter bag group vs 7(12%) for underwater seal. This similarity should be interpreted with caution, due to the larger sample size in the Graham *et al.* study compared to this study.

## Conclusion

Malignant and paramalignant pleural effusions occur more in females with breast cancer and majority of these patients are in the fourth and fifth decades of life. The flutter valve drainage bag is an effective and safe alternative to the standard underwater seal drainage bottle in the management of non-massive malignant and paramalignant pleural effusion. The flutter bag drainage system encourages earlier mobilization of patients when compared to the underwater seal drainage bottle and shortens the length of hospital stay. However, a multicentre study is needed to further validate the findings of this study.

### What is known about this topic

- The flutter valve drainage bag can be used to manage patients with pneumothorax and persistent air leak;
- The Flutter valve drainage bag can be used as a postoperative chest drain.

### What this study adds

- The use of the flutter valve bag can be extended to patients with non-massive malignant or paramalignant pleural effusion;
- This study also shows that the flutter valve drainage bag shortens in-hospital stay.

## Competing interests

The authors declare no competing interests.
